# The Effects of Positive Affect and Episodic Future Thinking on Temporal Discounting and Healthy Food Demand and Choice Among Overweight and Obese Individuals: Protocol for a Pilot 2×2 Factorial Randomized Controlled Study

**DOI:** 10.2196/12265

**Published:** 2019-03-20

**Authors:** Sara M Levens, Sara J Sagui-Henson, Meagan Padro, Laura E Martin, Elisa M Trucco, Nina A Cooperman, Austin S Baldwin, Angelos P Kassianos, Noreen D Mdege

**Affiliations:** 1 University of North Carolina at Charlotte Department of Psychological Science Charlotte, NC United States; 2 Osher Center for Integrative Medicine University of California, San Francisco San Fransisco, CA United States; 3 Department of Psychological Science University of North Carolina at Charlotte Charlotte, NC United States; 4 Hoglund Brain Imaging Center University of Kansas Medical Center Kansas City, KS United States; 5 Department of Psychology Florida International University Miami, FL United States; 6 Department of Psychiatry Rutgers Robert Wood Johnson Medical School Piscataway, NJ United States; 7 Department of Psychology Southern Methodist University Dallas, TX United States; 8 Department of Applied Health Research University College London London United Kingdom; 9 University of York Department of Health Sciences York United Kingdom

**Keywords:** obesity, cancer, temporal discounting, food choice, eating behavior, episodic future thinking, positive affect, guided imagery, randomized controlled trial

## Abstract

**Background:**

Unhealthy behaviors (eg, poor food choices) contribute to obesity and numerous negative health outcomes, including multiple types of cancer and cardiovascular and metabolic diseases. To promote healthy food choice, diet interventions should build on the dual-system model to target the regulation and reward mechanisms that guide eating behavior. Episodic future thinking (EFT) has been shown to strengthen regulation mechanisms by reducing unhealthy food choice and temporal discounting (TD), a process of placing greater value on smaller immediate rewards over larger future rewards. However, these interventions do not target the reward mechanisms that could support healthy eating and strengthen the impact of EFT-anchored programs. Increasing positive affect (PosA) related to healthy food choices may target reward mechanisms by enhancing the rewarding effects of healthy eating. An intervention that increases self-regulation regarding unhealthy foods and the reward value of healthy foods will likely have a greater impact on eating behavior compared with interventions focused on either process alone.

**Objective:**

This study aimed to introduce a protocol that tests the independent and interactive effects of EFT and PosA on TD, food choice, and food demand in overweight and obese adults.

**Methods:**

This protocol describes a factorial, randomized, controlled pilot study that employs a 2 (affective imagery: positive, neutral) by 2 (EFT: yes, no) design in which participants are randomized to 1 of 4 guided imagery intervention arms. In total, 156 eligible participants will complete 2 lab visits separated by 5 days. At visit 1, participants complete surveys; listen to the audio guided imagery intervention; and complete TD, food demand, and food choice tasks. At visit 2, participants complete TD, food demand, and food choice tasks and surveys. Participants complete a daily food frequency questionnaire between visits 1 and 2. Analyses will compare primary outcome measures at baseline, postintervention, and at follow-up across treatment arms.

**Results:**

Funding notification was received on April 27, 2017, and the protocol was approved by the institutional review board on October 6, 2017. Feasibility testing of the protocol was conducted from February 21, 2018, to April 18, 2018, among the first 32 participants. As no major protocol changes were required at the end of the feasibility phase, these 32 participants were included in the target sample of 156 participants. Recruitment, therefore, continued immediately after the feasibility phase. When this manuscript was submitted, 84 participants had completed the protocol.

**Conclusions:**

Our research goal is to develop novel, theory-based interventions to promote and improve healthy decision-making and behaviors. The findings will advance decision-making research and have the potential to generate new neuroscience and psychological research to further understand these mechanisms and their interactions.

**Trial Registration:**

ISRCTN Registry ISRCTN11704675; http://www.isrctn.com/ISRCTN11704675 (Archived by WebCite at http://www.webcitation.org/760ouOoKG)

**International Registered Report Identifier (IRRID):**

DERR1-10.2196/12265

## Introduction

### Background and Rationale

Unhealthy behaviors, such as poor food choices and physical inactivity, are associated with numerous negative health outcomes, including, but not limited to, multiple types of cancer and cardiovascular and metabolic diseases [1]. Notably, these behaviors can contribute to weight gain and obesity, which remains one of the top preventable causes of morbidity and mortality worldwide [2,3]. Only 9-13% of US adults met fruit and vegetable intake recommendations (ie, 1.5 to 2 cups of fruits a day, 2 to 3 cups of vegetables a day) in 2013 [4]. Similarly, in England, only 26% of adults consumed 5 or more portions of fruits and vegetables a day in 2016 [5]. Interventions targeting diet may help individuals lose weight in the short term, but often have minimal impact on weight loss maintenance, potentially because they do not target the underlying cognitive and affective mechanisms of eating behavior [6].

Eating behavior is a complex process regulated by homeostatic, physiologic-driven mechanisms that drive eating in response to hunger [7] and nonhomeostatic, reward-driven mechanisms that drive eating in response to highly palatable external cues (eg, foods high in fat and sugar) [8]. The rewarding nature of highly palatable foods can lead to eating in the absence of hunger, and in turn, overeating can result in increased reward responsivity to certain foods and subsequent weight gain [9]. Dual-system neural models of eating behavior theorize that the increased neural reward responsivity can be mitigated by activating areas of the brain associated with regulation to dampen hyperactive reward responses and manage unhealthy eating behaviors [10]. The regulation network, referred to as the *executive* [11], *deliberative* [12], or *reflective* system [13], includes brain regions associated with cognitive control, emotion regulation, and goal-directed behavior. In contrast, the reward network, referred to as the *impulsive* [11,13] or *automatic* [12] system, includes brain regions associated with evaluating, anticipating, and processing rewards. When there is an imbalance between these networks, the reward network can override the regulation network, whereby overeating leads to weight gain and eventually obesity [14]. Thus, among obese individuals, the regulation network is often considered underactive and the reward network is considered overactive [14].

To promote the healthy diet of fruits, vegetables, legumes, nuts and whole grains, and minimal sugars and fats recommended by the World Health Organization [15], healthy eating interventions should build on the dual-system model described above to target both the regulation and reward mechanisms that guide eating behavior. One regulation mechanism relevant to food choice is delayed gratification—the ability to resist the temptation of an immediate reward (eg, highly palatable food) in preference of a later reward (eg, long-term health) [10]. Prior research suggests that individuals who are obese show poor delayed gratification and demonstrate greater temporal discounting (TD), meaning they place greater value on smaller immediate rewards over larger or delayed rewards in the future [16-18]. For example, an individual with a low delay of gratification may value the satisfying taste of savory or sweet food that is available now, over the health benefits of future weight loss. Prior work indicates increased brain activation in regulation regions when participants make decisions involving delayed rewards and increased activation in reward regions when participants make decisions involving immediately available rewards [19]. Decreasing TD may, therefore, make it easier to favor the long-term reward of making a healthy food choice over the immediately available taste reward that may be associated with unhealthy food choice.

Multiple studies have demonstrated that the cognitive process of episodic future thinking (EFT; ie, the ability to imagine or simulate personal experiences that might occur in one’s future) reduces TD, especially in overweight and obese individuals [20]. Research delving into the mechanisms of EFT suggests that EFT is derived from episodic memory, which supports future simulation by allowing people to flexibly retrieve and recombine elements of past experiences into novel representations of events that might occur in the future [21]. Evidence from thought sampling procedures indicates that episodic future thoughts occur frequently in everyday life and serve a range of functions, including decision making, emotion regulation, intention formation, and planning [21]. Furthermore, training in EFT (vs episodic recent thinking [ERT]) has been shown to reduce discounting rates and food reinforcement [22] as well as behavioral outcomes such as calorie consumption [23,24]. These lab studies and pilot interventions suggest that EFT has the capacity to reduce TD and calorie consumption; yet, these interventions do not target the reward mechanisms that impact eating decisions. In addition, targeting the reward mechanisms involved in food choice could strengthen the impact of an EFT-anchored intervention.

One way to target the reward mechanisms involved in obesity is by enhancing the rewarding effects of healthy eating and creating positive associations with healthy food. Descriptive and observational research has shown that positive affect (PosA) is associated with healthier food [25,26], but this association may be bidirectional, with some studies showing fruit and vegetable consumption predicting PosA [27,28]. Affect is the representation of the body’s core evaluation, at any level and in any modality (including physiological reactions, emotions, thoughts, and expressions), that an object, event, or person encountered in the world is good for it, bad for it, approachable, or avoidable [29]. Accordingly, PosA is the physiological and emotional experience that an object is beneficial and confers positive value to the body and self. Importantly, a study experimentally manipulating PosA showed that creating positive associations with fruit (as opposed to neutral or negative associations) significantly increased the likelihood of choosing fruit in a behavioral choice task [30]. Consistent with the dual-system model, eating behavior interventions may need to take advantage of the overactive reward system that has been associated with overeating and obesity by enhancing the rewarding associations with healthy eating and its effects.

PosA has also been shown to increase TD [31], indicating that EFT exercises and feelings of PosA may have an interactive or additive effect on discounting rates. Both mechanisms have different underlying neurological pathways, and their interactive effect has not been tested to date. Although programs and interventions to increase healthy food choices exist, new interventions firmly grounded in behavioral, cognitive, affective, and neuroscientific theory may have a stronger impact on increasing healthy food choices than existing interventions. An intervention focused on enhancing both reward for healthy foods and regulation for unhealthy foods is likely to have a greater impact on dietary choices compared with interventions focused on either process alone.

### This Study

This protocol aims to test whether an intervention focused on enhancing both the reward value of healthy foods and regulation surrounding unhealthy foods is likely to have a stronger effect on eating behavior compared with interventions focused on either process alone. To achieve this, we will conduct a 2×2 factorial, randomized, controlled lab-based intervention study of brief guided imagery exercises that target regulation (EFT: yes, no) and reward (PosA imagery: positive, neutral) mechanisms of eating behavior.

To assess the individual and combined effects of EFT and PosA on eating behavior, we will examine the effect of PosA, EFT, and their interaction on (1) TD, (2) the reward value of healthy and unhealthy foods (ie, food demand indexed by intensity or the number of items consumed when freely available), and (3) food choice. Our predictions are 3-fold. First, we predict that participants in the EFT condition will demonstrate lower TD, lower demand for unhealthy foods, higher demand for healthy foods, more healthy food choices, and less unhealthy food choices compared with the ERT condition. Second, we predict that participants in the PosA condition will demonstrate no differences in TD, yet exhibit lower demand for unhealthy foods, higher demand for healthy foods, more healthy food choices, and less unhealthy food choices compared with the neutral affect conditions. Third, we predict that participants in the EFT and PosA conditions will demonstrate the lowest TD, the lowest demand for unhealthy foods, the highest demand for healthy foods, the healthiest food choices, and the least unhealthy food choices compared with participants in all other conditions.

## Methods

### Study Design

**Figure 1:**
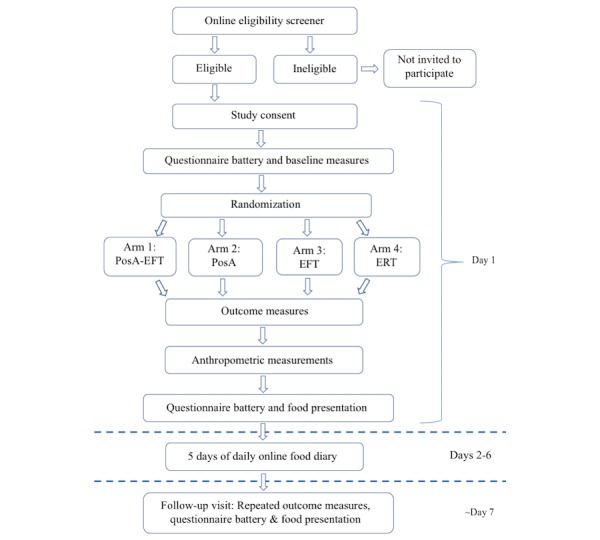
Flow diagram of study procedures. PosA: positive affect; EFT: episodic future thinking; ERT: episodic recent thinking.

This study is a factorial, randomized, controlled pilot study that employs a 2 (PosA imagery: positive, neutral) by 2 (EFT: yes, no) design in which participants are randomized to 1 of the 4 arms. Figure 1 shows the flow diagram depicting the experimental design. The 4 intervention arms are listed below:

1. Arm 1: PosA-EFT
2. Arm 2: PosA and *no* EFT
3. Arm 3: neutral affect and EFT
4. Arm 4: neutral affect and *no* EFT (ERT control condition)

### Sample Size

The target sample size of 156 participants (39 participants per group) is based on 2 prior studies testing EFT interventions. Sze et al [22] found that Web-based training in EFT (vs ERT) reduced TD with an effect size of *η*^2^=0.18. O’Neill et al [23] found that smartphone-delivered training in future (vs recent) episodic thinking impacted food choice by reducing total calorie consumption (*η*^2^=0.28) and percent calories consumed from fat (*η*^2^=0.28). We plan to use a mixed-design analysis of variance (ANOVA) with treatment arm (4 arms) as the between-subject variable and measurement period (baseline, after allocation, and closeout) as the within-subjects variable. As the EFT cue utilized by O’Neill et al was personalized to each participant and was delivered repeatedly via smartphone, and this protocol includes none of those design features (the intervention content is standardized and delivered once), we predict a smaller effect size. To be conservative, we predict the interaction between time and intervention arm variables will yield a standardized effect size of 0.17. Attrition rates commonly observed in brief guided imagery intervention studies are approximately 20% [32,33]. Therefore, we aim to recruit 156 total participants (39 participants per group) to allow for an analytic total sample of 124 participants with 31 participants in each group. This would provide 95% power to detect a statistically significant interaction between intervention arm and measurement period at the .05 level while adjusting for attrition.

### Participants and Setting

Participants will be recruited from the Charlotte, North Carolina, area community and asked to attend 2 in-person lab sessions separated by 5 days of daily diet tracking. The study will take place in a research lab in the Department of Psychology at the University of North Carolina at Charlotte, with a target recruitment of approximately 50% female. The United States Census Bureau provides estimates about the population characteristics in each state. Our race and ethnicity recruitment targets are derived from the Charlotte, NC 2017 Census [34]. To represent the Charlotte, NC population, we aim to recruit approximately 43% non-Hispanic white, 35% African American, 13% Hispanic or Latino of any race, 6% Asian American, and 3% other race (eg, Native Hawaiian or other Pacific Islander) or 2 or more races.

### Study Procedures

#### Identification

Potential participants will be recruited through postings in classified advertisement websites and on Facebook forums, through flyers posted at community buildings such as libraries and grocery stores, and through recruitment advertisements sent through university listservs reaching students, faculty, and staff. All individuals receiving this recruitment information will be considered potential participants.

#### Eligibility Assessment

Eligibility will be determined before coming to the lab by a Web-based screening questionnaire delivered via the Qualtrics survey platform. This survey will include basic demographic questions as well as self-reported height and weight (to calculate inclusion body mass index [BMI]) and questions assessing exclusionary medical conditions that could confound dietary choices (see below).

Participants are eligible if they:

1. Are aged between 18 and 63 years. We set the upper age limit at 63 years to limit the potential confounding effect of older age. Older adults may show a greater preference for immediate over delayed rewards [35], and this preference may be stronger among older adults with mild cognitive impairment [36-38] and Alzheimer disease [37,38]. On the other hand, other studies indicate that older adults may show a greater preference for delayed rewards compared with middle-aged adults [36]. In sum, TD may shift in older individuals as there is potentially less time in the future. Hence, our upper age limit is set at 63 years to minimize this age-related shift.
2. Have a BMI indicative of overweight or obese status (BMI≥25 kg/m^2^), measured first through self-report and then verified at the baseline visit with anthropometric measurements.

Participants are ineligible if they:

1. Report any conditions that could affect their food choices (ie, special dietary conditions, including diagnoses of celiac disease or type 1 diabetes; are currently being treated for an eating disorder; have ever had gastric bypass surgery; are allergic to nuts or peanuts; or practice a vegan diet) or responses during the lab tasks.
2. Have any devices (eg, pacemaker) in their body that could be disrupted by the bioimpedance scale measuring body fat percentage.
3. Are currently pregnant.

#### Consent Procedure

Informed consent for the study will be obtained upon arrival at the study lab in a face-to-face interaction with trained research staff. The research staff will review study information with the participant to ensure good comprehension and understanding and to answer any questions for clarification. A waiver to allow withholding information about several portions of the study procedure during consent was obtained from the ethics board. First, we will not inform the participants that we will be recording their selection of unhealthy and healthy snacks. Disclosure of this portion of the study may bias participants to not select food naturally. Second, we will not be disclosing the BMI inclusion and exclusion criteria, as disclosure could significantly increase the prospective participants’ emotional distress as they could learn that they meet criteria for obese or overweight status. Third, we will not disclose inclusion and exclusion dietary health conditions that could impact food choice (eg, gluten intolerance, celiac disease, or type 1 diabetes). Finally, to ensure that participants are blind to intervention condition assignment, the consent form will not disclose the overall factorial design of the study, the number of intervention arms, or the intervention content.

### Ethics and Confidentiality

The study has been granted ethical approval by the Health Sciences Research Governance Committee, University of York (December 4, 2017), and the University of North Carolina at Charlotte Institutional Review Board (17-0388; October 6, 2017). A signed copy of the informed consent will be kept by the research staff, and a copy is available to the participants for their records.

### Randomization Process and Blinding

All questionnaires and intervention content will be delivered via the Qualtrics Web-based survey platform. After the consent is obtained, randomization will be achieved via the Qualtrics software randomizer function. Upon starting the experimental session on the computer, the Qualtrics survey will randomly deliver 1 of the intervention arms to each participant without any input needed from the researcher, resulting in double blinding. The consent information will only state that the participants will listen to a short recording instructing them to think about physical sensations or future events. There is no information provided in the consent form on the study design, and there is no mention of PosA, EFT, or the number of intervention arms. Therefore, both the researcher and the participant will be unaware of the intervention arm to which the participant is assigned. The Qualtrics randomization procedure will evenly present the 4 intervention arms or guided mental imagery schemes across participants.

### Intervention Content

Initial scripts for the interventions were developed and then presented to an advisory panel of professionals who are experts in guided mental imagery stimuli, affect manipulation, future episodic thinking, and eating from the United Kingdom and United States. Guided imagery scripts were also presented to 2 face-to-face community advisory panels, one in the United States and the other in the United Kingdom. The intervention development process is described in detail elsewhere [39].

During the guided imagery including PosA (arm 1 [PosA-EFT intervention] and arm 2 [PosA intervention]), participants will be invited to think of positive feelings and associations surrounding healthy fruits and vegetables. They will be asked to imagine themselves appreciating the healthy food and to bring awareness to the positive aspects of the food, such as its color, feel, smell, taste, and health benefits. Additionally, participants in the PosA-EFT intervention (arm 1) will then be invited to think in detail of a future where they have made healthy food choices. The EFT intervention (arm 3) focuses on future-oriented thinking as described above, but includes no positive emotions surrounding healthy food. Finally, the ERT intervention control (arm 4) asks participants to think about a recent event and aims to rule out potential effects of guided imagery alone on food decision-making and TD and to control for the effort involved in imagining an event at a different time than the present. The interventions will be delivered during the first experimental session (see the Experimental Sessions section below) using Qualtrics survey software to play the audio recording of the guided imagery for each of the 4 intervention arms.

### Quality Assurance of Treatment Delivery

The interventions are audio recordings that the participants will listen to during the first experimental session. Thus, they are standardized across all participants to ensure the consistent delivery of each intervention condition. After the intervention is delivered, 3 questions will be asked that function as intervention manipulation checks. Immediately after the intervention, participants will be asked to describe some of the thoughts that came to their minds while listening to the guided imagery recording. Next, participants will be asked to rate how positive and negative they felt *during the guided imagery* on a scale from 1 (“not at all”) to 10 (“extremely”). Participants’ written text responses, describing their thoughts during the intervention, will be blindly coded for the occurrence of PosA, EFT, and recent past episodic thinking.

### Training

A large team of approximately 6 research assistants, led by 1 or 2 trained lead research assistants, will implement the experimental procedures. A protocol manual will describe the entire experimental session and include information on how to guide the participant through the questionnaires and body measurements. Each research assistant will be trained multiple times in implementing the protocol. In addition, each assistant’s first scheduled participant session, for each time point, will be observed by 1 of the lead research assistants. Finally, follow-up drop-in observations will be scheduled periodically to maintain consistently accurate data collection.

### Experimental Sessions

The study protocol includes 2 in-lab experimental sessions separated by approximately 5 days of at-home daily food diary entries (see Table 1). All questionnaires, intervention content, and baseline and outcome measures are delivered via the Qualtrics Web-based survey platform. Participants will be asked to fast for 2 hours before each experimental session, and all sessions will be scheduled after 10:00 am.

During the first experimental session, the participants will complete a battery of questionnaires, a baseline TD task, and a baseline food demand task (see the Primary Outcome Measures section below). Then, they will be randomly assigned to receive 1 of the 4 guided imagery intervention arms followed by manipulation check questions. Next, they will complete the TD and food demand tasks again, after which they will be offered a variety of snack foods to assess food choice. Food items will be placed next to the participants while a research assistant reads a standardized food choice script. At the end of the session, the participants’ anthropometric measurements will be taken, they will be scheduled for the second experimental session, and they will be instructed on when and how to complete the daily food diaries. The duration of the first session will be approximately 90 min, and each daily food diary entry will take approximately 5 min to complete. During the second experimental session, participants will complete the TD and food demand tasks again. They will be asked to complete another battery of questionnaires while they are again presented with a variety of snacks to eat, to assess their food choices a second time. The duration of the second session will be approximately 40 min. The schedule of enrollment, interventions, and assessments for the study is shown in Table 1.

### Reimbursements

Participants will receive US $10 for completing the first experimental session, US $5 for completing at least 3 of the 5 food diaries, and another US $10 for completing the second experimental session, to compensate for potential participation-related expenses. Participants who do not complete all parts of the study (eg, they do not return for the second session) will only be reimbursed for the parts of the study they completed. Participants who complete all parts of the study will also be entered in a random drawing for a chance to win 1 of the 2 Target gift cards worth US $100.

**Table 1:** SPIRIT Figure**:** Schedule of enrollment, intervention, and assessments.

Time point		Study period							
		Recruitment	Enrollment and allocation	Postallocation daily food surveys					Close out
		−t_1_	0	t_1_	t_2_	t_3_	t_4_	t_5_	t_6_
**Enrollment**									
	Eligibility screen	X	—^a^	—	—	—	—	—	—
	Informed consent	—	X	—	—	—	—	—	—
	Allocation	—	X	—	—	—	—	—	—
**Interventions**									
	Positive affect and episodic future thinking	—	X	—	—	—	—	—	X
	Positive affect	—	X	—	—	—	—	—	X
	Episodic future thinking	—	X	—	—	—	—	—	X
	Neutral affect and episodic recent thinking	—	X	—	—	—	—	—	X
**Assessments**									
	Baseline: TD^b^	—	X	—	—	—	—	—	—
	Baseline: Food demand	—	X	—	—	—	—	—	—
	Outcome: TD	—	X	—	—	—	—	—	X
	Outcome: Food demand	—	X	—	—	—	—	—	X
	Outcome: Food choice	—	X	—	—	—	—	—	X
	Outcome: Daily food surveys	—		X	X	X	X	X	—
	Assessments before allocation^c^	—	X	—	—	—	—	—	—
	Assessments after allocation^d^	—	X	—	—	—	—	—	—
	Assessments at close out^e^	—		—	—	—	—	—	X
	Repeated assessments (before allocation and at close out)^f^	—	X	—	—	—	—	—	X

^a^—: not applicable.

^b^TD: temporal discounting.

^c^Assessments before allocation: food frequency, reward-based eating, health-specific self-efficacy, weight related eating, health locus of control, perceived stress reactivity.

^d^Assessments after allocation: emotion regulation, trait mindfulness, behavioral motivations, impulsiveness, mental health history, physical health history, demographics.

^e^Assessments at closeout: global self-reported health, self-reported weight status, social desirability, coping responses, tobacco and alcohol use, sleep quality, loneliness, psychological flexibility.

^f^Repeated assessments (before allocation and at closeout): stages of change in weight management; *Covariate measures included in analyses*: perceived stress, positive and negative affect, and depressive symptoms.”

### Primary Outcome Measures

#### Temporal Discounting

The minute monetary TD task will be given at baseline, after allocation, and at closeout to assess TD. During the minute monetary TD task, participants answer 5 questions about their preference to receive specific monetary rewards over time (now vs later). Across consecutive trials, participants are presented with a fixed set of choices between smaller, immediate rewards and larger, delayed rewards (US $1000 in 3 weeks or US $500 now) with the temporal distance being adjusted at each trial (now, 4 days, 1 week, 3 months, and 2 years). The 5 questions are taken from a list of 64-item pairs with differential money and time options. The questions adjust based on the participants’ prior response to identify their delay discounting rate. For example, the participants select which option they would rather have “US $500 now” or “US $1000 in 3 weeks.” If the “US $1000 in 3 weeks” is selected, the next question will feature the same monetary reward at a more distant time (ie, “US $1000 in 1 year”). However, if the “US $500 now” option is selected, the next question will feature the US $1000 reward at a more proximal time (ie, “US $500 now” or “US $1000 in 1 day”). In this way, the reward amount or time will be titrated to identify the rate (ie, k-value) at which the participant discounts monetary rewards over time. The TD rate will be calculated for each participant and then compared across treatment group as a between-subjects variable and as a within-subjects repeated measure variable, comparing the effect of the treatment (baseline compared with after allocation and at closeout).

#### Food Demand

The food demand task will be given at baseline, after allocation, and at closeout. It is based on a food purchasing task [37] that assesses the amount participants are willing to pay (ie, reward value) for different quantities of snack foods. Participants will first view photos of different healthy (eg, apple, yogurt, and popcorn) and unhealthy (eg, potato chips, chocolate chip cookies, and candy bar) snack foods that correspond with the real food items they are presented with in the food choice task (see the Food Choice section below). Participants select their most preferred healthy food and their most preferred unhealthy food from the list and answer the questionnaire for each of the 2 food items. These preferred food photos are displayed on the computer during the task so that participants can refer to them when completing the task. The instructions, modified from the study by Epstein et al [40], are available in Multimedia Appendix 1.

Food demand task responses will be used to generate a food demand curve, reflecting the quantitative relationship between demand for food and escalating price. We will calculate the food demand curve for each of the preferred healthy and unhealthy options. Calculating the food demand curve generates 5 indices: (1) food demand breakpoint (ie, the first price at which consumption was 0), (2) intensity of food demand (ie, consumption at the lowest price), (3) elasticity of food demand (ie, sensitivity of snack food consumption to increases in cost), (4) Omax (ie, maximum expenditure for snack food), and (5) Pmax (ie, price at which expenditure was maximized). Calculating the food demand curve for each of the preferred or chosen healthy and unhealthy options will give rise to 10 values (5 indices for each healthy and unhealthy food categories). The primary index of demand will be intensity of food demand, which will be compared across treatment group as a between-subject variable and as a within-subject repeated measure variable, comparing the effect of the treatment (baseline compared with after allocation and at closeout). However, all indices will be calculated to fully describe the demand curve.

#### Food Choice

Food choice will be assessed by presenting participants with an array of healthy and unhealthy snack options after allocation and at closeout. Participants will be presented with the snack options on a tray, and all items will be presented as pairs (ie, 2 apples, 2 bags of chips, or 2 candy bars) to encourage selection. Participants will be presented with a total of 9 healthy and 9 unhealthy snack options (excluding drink options). Importantly, 2 of each snack option will be presented, so participants will see a total of 18 healthy and 18 unhealthy snack items (36 total snack items). Healthy snack food items have a total calorie count that ranges from 35 to 180 calories per portion. Unhealthy snack food items have a total calorie count that ranges from 230 to 330 calories per portion. An attempt was also made to match items categorically, for example, an unhealthy option such as potato chips will be provided along with a healthy option such as low-calorie popcorn and rice puffs. The snack items will be arranged neatly on the tray and will resemble the snack tray that is sometimes presented to guests in a hotel room. Alongside the tray, participants will be presented with accompanying unhealthy (soda) or healthy (water) drink choices. As with the snack, 2 of each drink option will be available.

Research assistants will describe the availability of the snack options in the context of the requirement that participants fast before the study appointment. This approach is expected to reduce potential participant bias that could occur if participants are aware their food choices will be recorded. The following is a section of the script that research assistants will deliver at this time:

In our lab, we always ask participants not to eat or drink anything before coming into the lab to make sure that differences in how hungry you are or the time since your last meal doesn’t affect your choices on the tasks or your preferences. We know it can be hard to fast and since we ask participants to not eat for two hours before coming in, we like to give people a range of snacks to eat.

Participants will then be encouraged to select any snack option to eat during the session (while completing the final batch of questionnaires) or take the food(s) with them to eat later at their leisure. The research assistant will then leave the participant alone with the snack options while they complete the remaining questionnaire items. All food choices will be recorded by the research assistant after the participant departs. The recorded number of healthy and unhealthy snacks will be summed, respectively, to create *healthy food choice* and *unhealthy food choice* variables for each participant. These variables will be compared across treatment group as a between-subject variable and as a within-subject repeated measure variable, comparing the effect of the treatment (baseline compared with after allocation and at closeout).

#### Daily Food Diaries

To assess food choice during the 5 days between allocation and closeout, participants will be sent, by email, a link to complete a daily Web-based food diary. This questionnaire is based on the Paffenbarger Physical Activity Questionnaire Dietary Habits subscale [41] and other food frequency questionnaires. Participants will be asked to complete the survey at the end of the day when they do not plan on eating anything afterward. The survey will first ask the participants to briefly describe and/or list the food items or meals they ate that day. Then, participants will be asked to report the number of servings they ate that day for different food categories. The food categories will include fruits; vegetables; grains; eggs; milk and cream; dairy (not including milk); poultry; fish and seafood; beef, pork, lamb, and other red meat; nuts, seeds, and legumes; fats and oils; sweets and desserts (not including candy); candy; salty snacks; drinks; and other foods. For each food category, several examples will be provided to describe what equates to 1 serving of that category. The reported number of daily servings for each of the food categories will be averaged to create a measure of outside-the-lab food intake. For analysis, outside-the-lab food intake will be compared across treatment groups to assess the impact of the intervention arm on outside-the-lab food choices.

### Demographic and Anthropometric Measures

#### Demographic Information

Participants will self-report their age and biological sex (0=male, 1=female). They will also report their relationship status, education, and income group that best represents themselves from preselected options. Finally, they will be asked to report their racial and ethnic identity by indicating if they identify as Hispanic or Latino and select the racial group that best represents themselves from preselected options.

#### Anthropometric Measurements

During the first experimental session, height will be measured with a stadiometer and weight will be assessed with an electronic weight scale. Recorded height and weight will be used to confirm the participant’s self-reported height and weight on the screening questionnaire. Waist circumference will be measured with a tape measure. Body fat percentage will be assessed with an Omron bioimpedance scale. Blood pressure will be measured with an aneroid sphygmomanometer, Omron blood pressure monitor. Three successive arterial blood pressure readings will be taken on the participant’s left arm, with a 2-min interval between each reading.

### Covariates

Covariates thought to impact eating behavior will be assessed before allocation and 1 week later at closeout. Covariate measures are indicated in Table 1 in footnote f.

#### Perceived Stress

To control for the impact of stress on eating behaviors, we will measure self-reported perceived stress using the 14-item Perceived Stress Scale (PSS) [42]. The PSS assesses the extent to which situations in one’s life are appraised as stressful during the past month on a 5-point Likert scale (0=never, 4=very often).

#### Positive and Negative Affect

We will use the 10-item Positive and Negative Affect Scale (PANAS) [43] to control for the impact of dispositional affect on eating behaviors. The PANAS assesses the extent to which the individual felt positive or negative emotions in the past month. Participants rate their affect using a 5-point Likert scale ranging from very slightly or none at all to extremely. During the second experimental lab session, this questionnaire will be administered again, but instead ask about affect in the past week.

#### Depressive Symptoms

We will control for the impact of depression symptomatology on eating behavior by measuring depressive symptoms with the 10-item Center for Epidemiology Studies of Depression scale (CESD-10) [44]. The CESD-10 assesses mood symptoms during the past 7 days using a 4-point Likert scale.

#### Exploratory Measures

Several exploratory measures will be included in the study to determine if group assignment or our primary outcome measures are associated with psychological, behavioral, and emotional health constructs. Participants will complete a distinct set of exploratory assessments before allocation, after allocation, and at closeout (Table 1). Trait exploratory measures that are less likely to be impacted by the intervention content will be distributed after allocation and at closeout to also serve the practical design purpose of providing a consistent setting during which the participant is presented with the array of snacks that comprise the food choice measure. The description of each exploratory measure is provided in Multimedia Appendix 1.

### Analysis

#### Primary Statistical Analysis

Our primary statistical analyses will comprise a mixed design (between-subjects and repeated measure) that compares TD, food demand, and food choice measures at baseline, after allocation, and at closeout across treatment arms. Our analyses will control for standard demographic variables such as age, biological sex, race, and income. A series of repeated measure ANOVAs with treatment arm as a between-subject variable and measurement period as the repeated measure will be conducted on TD rates and healthy and unhealthy food demand indices, respectively.

In addition, to examine the effect of the intervention arms on food choices inside and outside the lab, a series of ANOVAs will be conducted on healthy and unhealthy snack food choices and average daily serving intake values for each of the food categories. To probe the effect of the intervention on in-lab food choices, we will conduct a repeated measure ANOVA with measurement period repeated across treatment group on health and unhealthy snack food choices. Next, to probe the effect of the intervention on food choices outside the lab, we will conduct a 1-way ANOVA with treatment arm entered as the between-subject variable on average daily serving intake values for the primary food categories (ie, fruit and vegetable intake).

#### Secondary Statistical Analyses

In exploratory analyses, we will examine correlations between summary scores on the exploratory questionnaire measures and outcome measures of TD, food demand, and food choice, to explore potential relationships between self-report and primary outcomes. For example, we plan to examine whether there is an association between change in primary outcome measures (from baseline to post allocation), physical activity, smoking, and alcohol use, to examine how these health behaviors influence the efficacy of the intervention. We also anticipate examining the association between trait mindfulness and changes in outcome measures, to determine if trait mindfulness influences intervention efficacy. In another potential exploratory analysis, we will test the association between trait impulsivity and changes in TD over the course of the study, to determine if high trait impulsivity is associated with greater stability in TD over time. These, and other analyses, will be used to explore the extension of the intervention to other health behaviors and for the development of future research questions and interventions.

## Results

Recruitment started on February 19, 2018, to begin feasibility testing of the protocol. Feasibility testing started on February 21, 2018, and continued through April 18, 2018, during which 20.5% (32/156) of the final sample was run through the protocol. As there were no major protocol changes required at the end of the feasibility phase, these 32 participants were included in the target sample of 156 participants. Recruitment, therefore, continued immediately after the feasibility phase. At the time this manuscript was submitted, participants are actively being recruited into this study, and 84 participants have completed the protocol. Recruitment is estimated to last approximately 10 months.

## Discussion

### Findings and Implications

The overall goal of this research is to develop novel, theory-based interventions to promote and improve healthy decision-making and behaviors. In this study, we focus on eating behaviors and test the overarching hypothesis that guided imagery interventions targeting PosA will increase the rewarding value of healthy foods, indexed by food choice and food demand, and guided imagery targeting EFT will increase regulation, indexed by TD. We predict a novel, synergistic effect between PosA associations toward healthy foods and positive EFT. Both mechanisms have different underlying neurological pathways, and their interactive effect has not been tested to date. Furthermore, we will test these interventions among individuals who are overweight or obese and may show the most benefit from the intervention because of a hypothesized overactive neural system of reward and underactive neural system of regulation. Thus, the findings will advance health behavior decision-making research. Moreover, given that the synthesis of affective and cognitive pathways is novel, the expected findings have the potential to generate new neuroscience and psychological research to further understand these mechanisms and their interactions.

Although the findings from this project will address basic mechanisms in the context of eating behavior, the effect of affective associations and future thinking likely translates across different health behaviors (eg, physical activity, substance use, and sun protection). Thus, developing brief manipulations of these 2 mechanisms (ie, PosA and positive EFT) holds great potential for future translation across multiple health behaviors. Moreover, given that the affective imagery and EFT manipulations are relatively brief and could be easily adapted to Web-based or smartphone app–based interventions, such interventions would be potentially scalable and wide-reaching. In fact, we expect that the findings from this project will directly inform future research targeting the 2 mechanisms (affective associations and future thinking). Moreover, these findings could inform research on existing eating behavior interventions, interventions for other behavioral domains, and interventions that could be Web-based or delivered through mobile phone apps.

### Strengths and Limitations

Despite the strengths of this study, some important limitations must be noted. As a pilot study, the sample will be small and will not have the power to examine individual differences in intervention effectiveness. It is also possible that some participants will have difficulty forming PosA associations with healthy food or that the formed positive associations will also extend to nonhealthy foods to increase the overall appeal of food in general. Although we do not expect this to be the case because the PosA-guided imagery stimuli have been designed to focus on the benefits of healthy food, a finding that PosA increases the appeal of food in general would regardless be helpful in designing guided imagery exercises that promote healthful eating. In addition, the guided imagery interventions differ in length—the PosA-EFT intervention arm with both PosA content and EFT content is longer than the other intervention arms as it needs to disseminate more content. It is possible that the difference in length across the intervention arms could affect the outcome measures; however, this confounder was preferable to shortening and potentially decreasing the efficacy of the PosA and future episodic content to match the lengths of the other intervention arms. Future research will be needed to test what guided imagery length is optimal.

With regard to the food choice outcome measure, it is also possible that participants may expect that their food choice is being recorded and they may monitor or alter their food choice to be in line with perceived expectations. Although we have designed our task instructions to mitigate this confounder, it is possible that biases in expectation may still exist as overweight and obese participants may feel that their food choices are being observed in any public setting. If so, the expectation bias may occur regardless of intervention arm; nevertheless, it is important to acknowledge that participants may not be making food choices in the lab as freely as they do in nonpublic settings. In addition, it is possible that our recruitment methods may affect our findings. Although we will recruit through university-based and community-based channels, we will not recruit through any weight loss–associated channels, which could affect our findings. Another limitation of this protocol is that we will not be able to stratify our randomization by biological sex; this will be an important limitation to address in future work.

### Conclusions

Despite these limitations, findings from this study have the potential to advance decision making, reward learning, and affective-cognition research as well as form the basis for potentially large-scale brief interventions that have the capacity to impact a range of health behaviors (eg, healthy eating, physical activity, substance use, and sun protection). Accordingly, findings will be disseminated at both basic and applied science conferences. In addition, the affective imagery and EFT manipulations are relatively brief and could be easily adapted to Web-based or mobile phone app–based interventions. If this study supports our hypothesis, then in the future, we will explore the feasibility, effectiveness, cost-effectiveness, and scalability of developing stand-alone and add-on interventions that manipulate PosA and EFT to promote healthy behavior.

## Appendix

Multimedia Appendix 1 Food demand instructions given to participants and description of all exploratory measures included in the study.

## Abbreviations

ANOVA: analysis of variance
BMI: body mass index
CESD: Center for Epidemiology Studies of Depression
EFT: episodic future thinking
ERT: episodic recent thinking
PANAS: Positive and Negative Affect Scale
PosA: positive affect
PSS: Perceived Stress Scale
TD: temporal discounting
