# Supplementary figures and images for: The Effects of Positive Affect and Episodic Future Thinking on Temporal Discounting and Healthy Food Demand and Choice Among Overweight and Obese Individuals: Protocol for a Pilot 2×2 Factorial Randomized Controlled Study
Source: JMIR Res Protoc. 2019 Mar 20;8(3):e12265. doi: 10.2196/12265 (PMC6446151; doi:10.2196/12265)

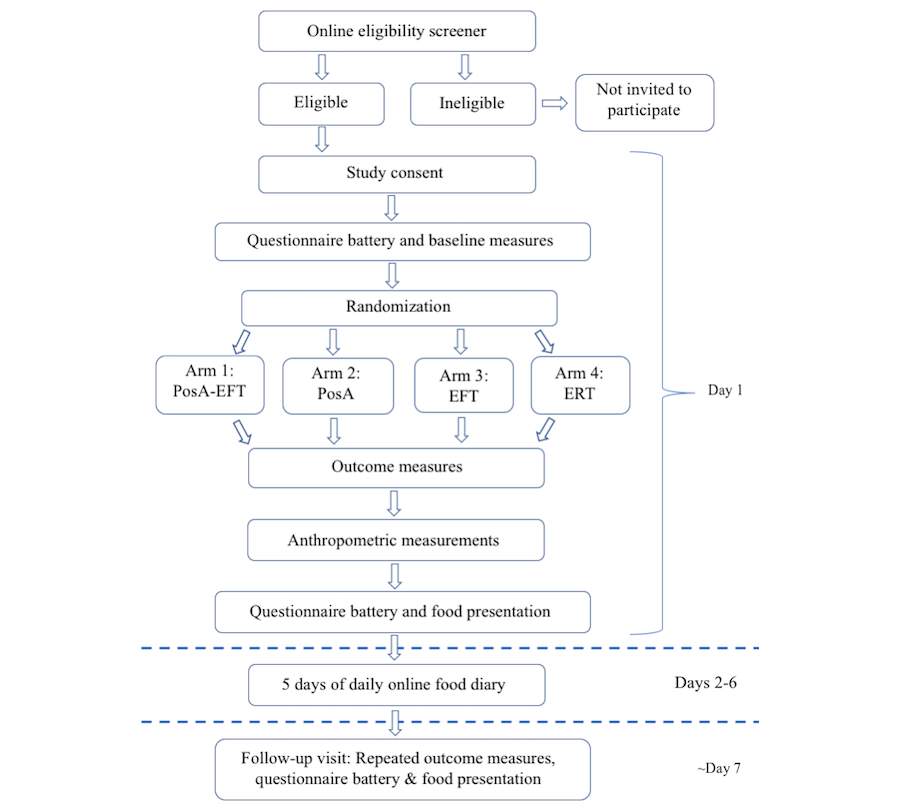

Supplement: Multimedia Appendix 1 [file resprot_v8i3e12265_fig1.png]
